# Research progress of aconitine toxicity and forensic analysis of aconitine poisoning

**DOI:** 10.1080/20961790.2018.1452346

**Published:** 2018-04-09

**Authors:** Xiangting Gao, Jun Hu, Xincai Zhang, Yuanyi Zuo, Yun Wang, Shaohua Zhu

**Affiliations:** a Department of Forensic Medicine, Soochow University, Suzhou, China;; b Laboratory of Biomedical Technology, Jiangsu Vocational College of Medicine, Yancheng, China;; c Department of Forensic Sciences, Binhai People’s Hospital, Yancheng, China

**Keywords:** Forensic sciences, aconitine, toxic effects, metabolic characteristics

## Abstract

Chinese herbal medicines have been extensively used in China and other countries for centuries. Aconitine, a diterpenoid alkaloid extracted from *Aconitum* plants, has anti-inflammatory and analgesic activities, but can also induce severe arrhythmia and neurotoxicity. Aconitine poisoning accidents caused by misuse, suicide, or homicide have been reported in recent years. In China, fatal aconitine poisoning can occasionally happen on account of accidental ingestion of some wild plants or consumption of herbal decoction made from the roots of *Aconitum* plants. However, it is rather difficult for forensic experts to find the specific results in present forensic autopsy of aconitine-induced death. To further clarify its potential risk following the widespread application of aconitine, toxicological characteristics and pharmacokinetics of aconitine are reviewed. Moreover, gastrointestinal, neurological, and cardiovascular symptoms were observed frequently in aconitine poisoning cases. In addition, the review also aims at providing some convincing evidences for forensic experts to identify unexplained death with postmortem examination.

## Introduction

Herbal medicines are commonly used in China and other countries to treat various diseases due to its natural, harmless and less adverse effects for thousands of years. Traditional Chinese medicines are processed by soaking or boiling before consumption to reduce the toxicity [1]. In general, herbal poisoning may frequently occur because of inadequate processing and preparation, overdose, contamination, misidentification, and even in some suicidal or homicidal cases. *Aconitum* plants have been extensively applied to treat multiple diseases in China and some other Asian countries [2–4]. Previous studies [5,6] on *Aconitum* plants have showed its pharmacological properties such as anti-inflammation, analgesia, and anti-rheumatism. Aconitine is one of the major bioactive alkaloids extracted from Monkshood (*Aconitum napellus*), a plant getting its name for its blue to purple coloured flowers that resembles a monk’s hood [7]. Aconitine belongs to the *Aconitum* genus of Ranunculaceae family and is frequently employed in herbal medicines for its anti-inflammatory, analgesic, anti-rheumatic, and cardiotonic actions [8–10]. In addition, aconitine could also suppress tumour growth and induce cell apoptosis by NF-ĸB signalling pathway in human pancreatic cancer, indicating that aconitine may serve as a potent therapeutic strategy for the treatment of several cancers [11]. However, the application of aconitine has been limited in clinical practice due to its toxic effects on the heart and nervous system.

With the increasing popularity of herbal drugs, herb-induced fatal poisoning cases have frequently happened as a result of inappropriate use of herbs in recent years. Aconitine, one of the abundant and high bioactive diterpenoid alkaloids, has a narrow therapeutic index, and it also brings great challenges to identify its appropriate dosage. As far as we know, aconitine poisoning cases occasionally happen in China and some other parts of Asia. For example, people are accustomed to making medicinal liquor or herbal decoctions containing aconitine for treating illness and enhancing health in some rural areas of China [12]. Thus, it is of great concern that improper process and use can lead to severe poisonous cases or even unexpected death. In addition, aconitine poisoning can also happen in some accidental ingestions, suicide or homicide cases. Previous studies [13,14] revealed the half-maximally lethal dose (LD50) aconitine for mice is 1.8 mg/kg by oral administration, and the minimum lethal dose of oral administration in humans is evaluated to be 1–2 mg. Monoester diterpenoid alkaloids (MDAs) are considered as hydrolysed products of diester diterpenoid alkaloids (DDAs). The LD50 of aconitine for mice by intravenous injection is approximately 38 times of its hydrolysates [15,16]. Symptoms of aconitine poisoning frequently appear within 2 h or even several minutes via oral ingestion, indicating that it can be easily absorbed with oral administration. It has been demonstrated that aconitine can also be absorbed into systemic circulation through the human skin, leading to fatal and non-fatal poisoning [17]. Furthermore, aconitine poisoning may present with abnormities symptoms of gastrointestinal, neurologic, and cardiovascular system [18]. However, there is no specific antidote and the current treatment is mainly based on the supporting therapies. Nevertheless, it was reported that there were some effective treatment methods or medicines to improve cardiac arrhythmias in aconitine-induced poisoning accidents including charcoal hemoperfusion, amiodarone, magnesium, and lidocaine [19–21].

On the one hand, aconitine is very unstable and decomposed easily in the human body. On the other hand, it is not detected routinely for common toxicology analysis in present forensic practice. Meanwhile, little attention is paid to aconitine poisoning in current clinical practice and medicolegal expertise. Consequently, it is worth noting that the history of use of herbal medication or wild plants should be taken into consideration in exploring unexplained death.

In this paper, we review the toxicological properties and pharmacokinetics of aconitine. We also attempt to describe the autopsy results so as to provide some references for dealing with unexplained death in present forensic practice.

## The pharmacokinetic studies and determination method of aconitine

The ester groups combining with C8 and C14 are considered to be primarily responsible for the high toxicity of aconitine, while hydrolysis of esters can reduce its toxicity dramatically [22]. Aconitine is well known for its pharmacology actions, but also recognized as a toxic ingredient that needs to be processed properly in order to use it safely for humans. Aconitine can be hydro-lysed to less toxic benzyl aconine and aconine derivatives [23]. Considering that the therapeutic dose and the toxic dose of aconitine is close, it is crucial to use safely as a medicine in clinical applications. Indeed, establishment of the pharmacokinetic parameters of aconitine would be essential to make it play better pharmacological effects in clinics. Cytochrome P450 (CYP450) enzymes, belonging to membrane-bound hemoproteins, are involved in approximately 80% of phase I metabolism of drugs and play important roles in oxidative metabolism of drugs and exogenous substances [24–26]. CYP3A has been found to be the dominant CYP450 expressed in both liver and intestine, and it principally catalyses phase I metabolism of various alkaloids in human intestine and liver microsomes [27]. Whether CYP450 enzymes are involved in aconitine metabolism has also been further investigated. It has been reported that aconitine is mainly metabolized by CYP3A and CYP1A1/2 into less toxicity derivatives in rat liver microsomes [28]. In addition, further studies showed that aconitine can be converted into several CYP-mediated metabolites in human liver microsomes, and isoforms of CYP including CYP 3A4/5 and 2D6 were primarily responsible for the metabolism of aconitine [29].

It should be noted that the detection of aconitine concentrations in body fluids plays a vital role in clinic and forensic toxicology analysis of suspected poisoning incidents. As aconitine can decompose or metabolize rapidly, it is also difficult to detect aconitine contents in human body fluids. In previous studies, some methods have been employed for detecting aconitine such as capillary electrophoresis, gas chromatography-mass spectrometry, and high performance liquid chromatography [30,31]. However, low selectivity and sensitivity have restricted the application of these methods to some extent. At present, liquid chromatography tandem-mass spectrometry (LC-MS/MS) has developed a valid and precise method to analyse aconitine contents in blood and urine samples [32–34]. Taking into account that aconitine is metabolized fast into several derivatives in animal and human models, accordingly, the findings of aconitine and primary metabolites in blood, urine, or in herb medicine samples together with the important clinical manifestations, will provide some indispensable evidences of aconitine poisoning in present forensic practice.

## Symptoms of aconitine poisoning and pathological changes in postmortem examination

Aconitine, which has a narrow therapeutic window, is considered to be the principal highly toxic DDA in *Aconitum* alkaloids. The incubation period between ingestion of aconitine and the onset of symptoms may be as short as several minutes, indicating that aconitine can be absorbed rapidly after oral administration by the upper gastrointestinal tract. It has been reported that aconitine can reach relatively high concentrations in the liver, kidney, and heart following oral intake of aconitine [35]. Patients with aconitine poisoning generally present with a series of gastrointestinal, cardiovascular, and neurologic symptoms [36,37]. The gastrointestinal features can be nausea, vomiting, diarrhoea, and abdominal pain. Neurologic symptoms include numbness in mouth and limbs, paraesthesia, central nervous system depression, respiratory muscle depression, convulsions, and seizures. Cardiovascular manifestations predominantly include hypotension, palpitations, chest pain, bradycardia, sinus tachycardia, ventricular tachycardia, and ventricular fibrillation. Furthermore, the severity of symptoms appears to be related to the doses and time of exposure to aconitine. In particular, aconitine is also quickly decomposed or eliminated with a short half-life [38]. Thus, it is difficult for forensic specialists to identify death caused by aconitine poisoning with postmortem examination.

The signs of asphyxia are observed at autopsy including facial and nail bed cyanosis. Foamy liquid can be seen in the endotracheal cavity. The postmortem examination may find haemorrhagic points distributed on the surface of the heart and lungs [39,40]. Moreover, the microscopic examination has been reported to reveal bilateral intrapulmonary haemorrhage and oedema, congestion of multiple organs, and bilateral pleural effusions in different degrees [41]. It is worth noting that eosinophilic granulocytes can also be observed in the lungs, liver, pancreas, spleen, and gastrointestinal mucosal tissue [42].

The results of toxicological analysis are rather important for forensic identification of aconitine poisoning. So far, LC-MS/MS is a sensitive and validated method to identify toxic plant alkaloids [43]. It is necessary for forensic pathologists to exclude the presence of physical injury and fatal diseases. Meanwhile, the results of toxicological analysis showed that aconitine and its derivatives were positive and other toxic substances and illegal drugs were negative in blood, gastric content, or urine samples of victims. In this situation, death was generally considered to be caused by aconitine poisoning.

## Toxic effects of aconitine

It is rather difficult to define precisely the therapeutic dose and toxic dose of aconitine because of its narrow therapeutic index. The cardiotoxicity and neurotoxicity caused by inadequate consumption of aconitine have been occasionally reported in recent years [42,44]. Therefore, the public should be warned of the risk of aconitine poisoning.

### Toxic mechanisms of aconitine in cardiac system

Previous studies have revealed that aconitine could induce various types of life-threatening arrhythmias such as bradyarrhythmia, nodal tachycardia, cardiac fibrillation, bidirectional ventricular tachycardia, and intraventricular block [45–47]. *In vivo* experiment using murine model has confirmed that aconitine could suppress the progression of systemic lupus erythematosus and attenuate the pathologic impairment [48]. Aconitine-induced arrhythmias were observed in isolated rabbit atria, and results showed that aconitine might directly stimulate sinus node and inhibit the propagation of impulses in some degree [49]. Emerging evidences indicated that aconitine could block the inactivation of voltage-dependent sodium channels at the resting membrane potential causing sustained Na^+^ influx, therefore leading to arrhythmia [50,51]. The aconitine-induced blockade of HERG and Kv1.5 in Xenopus laevis oocytes is considered to be one of the mechanisms of cardiac arrhythmias [52]. Moreover, in H9c2 cells and cultured neonatal rat cardiomyocytes, aconitine could produce the inhibition effect on ultrarapid-delayed rectifier K^+^ current in a time- and dose-dependent manner [53].

Ca^2+^ is one of the important second messengers and plays crucial roles in the process of cells signal transduction and electrical activity of myocardial cells [54–57]. Meanwhile, disruption of the intracellular Ca^2+^ homeostasis is an important mechanism of arrhythmic toxicity of aconitine [51]. It is well known that aconitine can induce abnormities of spontaneous beating rate, amplitude of spontaneous oscillations, intracellular Ca^2+^ signals, and increase the relative intracellular Ca^2+^ concentration in cultured primary cardiomyocytes, indicating that disruption of intracellular Ca^2+^ homeostasis may contribute to arrhythmic toxicity in aconitine-treated cardiomyocytes [58]. Calcium regulatory proteins, including NCX1, RyR2, DHPR, and SERCA2a in sarcoplasmic reticulum, are capable of maintaining intracellular calcium homeostasis in myocardial cells [59]. Previous publications have described that aconitine could damage myocardial cells, increase the expression of RyR2 and decrease the expression of NCX1, DHPR-α1 significantly, thus leading to the unbalance of intracellular calcium homeostasis [60]. The results of current research provided strong evidences that aconitine-induced arrhythmia was associated with intracellular Ca^2+^ signals and pre-treatment with aconitine reduced the sarcoplasmic reticulum Ca^2+^-ATPase expression, increased the expression of Na^+^/Ca^2+^ exchange in rat ventricular myocytes, which is consistent with the previous studies [61,62].

Connexin 43 (Cx43) is the principal gap junction protein in heart [63]. Previous studies have demonstrated that Ser368, a protein kinase C site, is involved in the regulation of gap junction function. It is well known that the Ser368 remains phosphorylation status under normal circumstances [64,65]. Aconitine could induce Cx43 and protein kinase C-a dephosphorylation and alter Ca^2+^ oscillation frequency, which probably is associated with cellular signal transduction, finally leading to cardiac toxicity in cultured neonatal rats cardiomyocytes [66].

More importantly, aconitine could also shorten action potential duration, reduce L-type calcium currents, thereby contributing to the proarrhythmic effects in human-induced pluripotent stem cell-derived cardiomyocytes. The effects of aconitine-caused arrhythmia and underlying mechanisms were further studied in human cardiomyocytes model [67]. In addition, it is noteworthy that the Na^+^–Ca^2+^ exchange (NCX) system plays a vital role in regulating cardiac contractility and electrical activity in different animal modes. KB-R9743 (KBR), a selective inhibitor of NCX in cardiac muscle, is capable of inhibiting aconitine-induced arrhythmias in guinea pigs and isolated ventricular myocytes [62,68].

Drug–DNA interaction is regarded as one of the reasons of the DNA damage of some drugs [69]. The cytotoxicities of aconitine were further investigated in rat myocardial cells H9c2. The results showed that aconitine could exhibit cytotoxic activities including promotion of the apoptotic rate, inhibition of the growth of myocardial cells, and interaction with DNA by intercalation and electrostatic binding, implying that the aconitine-induced cardiac toxicity and DNA injury are correlated in a certain degree [70]. Our study showed that aconitine could induce apoptosis of H9c2 cells at least in part via mitochondria-dependent apoptotic pathway [71]. However, the specific mechanism remains unknown and still needs further study.

### Toxic effects of aconitine on nervous system

The neurotoxic effects of aconitine have been researched in different cell types. For example, it was reported that aconitine could block neuromuscular transmission and cause depolarization in mice and frog skeletal muscles [72,73]. More importantly, aconitine was reported to decrease the amplitude and block end-plate potentials and nerve action potentials, which contributes to neuromuscular blockade accompanied by excessive presynaptic depolarization in the isolated phrenic nerve-diaphragm muscles of rats [74]. Interestingly, a piece of published report demonstrated that aconitine could suppress delayed rectifier K^+^ current in differentiated NG108-15 neuronal cells and alterations in action potentials caused by aconitine might be concerned with abnormal neuronal excitability [75]. Moreover, it has been confirmed that aconitine can induce epileptiform activity in rat neocortical and hippocampal slices with acute and extended excitatory effects [76,77].

### Other toxic effects of aconitine

Published studies have indicated that aconitine has evident toxic effects on the growth of embryos and morphogenesis in rat embryos cultured model. It has been reported that aconitine could cause cardiac defect, irregular somites, and brain malformation during the period of organ formation, suggesting that teratogenesis may be induced by aconitine in the process of embryonic development [78]. The long-term physiological effects of aconitine were also further investigated in mice by detecting the rectal temperature, body weight, and by electrocardiogram, and the results revealed that the toxicity of aconitine might be decreased accompanied by chronic administrations and evaluated metabolic activity of aconitine [79,80]. P-glycoprotein, encoded by the *MDR1* gene, is one of the important transporters in the apical member of mucosal cells in the intestine and excretes toxic substances into the intestinal lumen [81]. It should be stressed that aconitine could dramatically increase the P-glycoprotein levels in mice and LS174T and Caco-2 cells, concomitantly reducing acute toxicity of aconitine and other drugs toxicity by drug–drug interactions [82].

## Conclusions and future prospects

With the widespread use of Chinese herb medicines, herbs-induced poisoning may be frequently encountered in the world. *Aconitum* plants are used in China and some Asian countries for treatment of various common medical problems such as pains, rheumatoid arthritis, and cardiac disorders [83]. *Aconitum* alkaloids, mainly containing aconitine, hypaconitine, and mesaconitine, are important representatives derived from the roots of plants in *Aconitum* genus. Aconitine, a high bioactive diterpenoid alkaloid derived from *Aconitum* plants, has great medicinal value, but also can cause serious poisonous cases. Despite this, based on the accumulation of traditional processing experiences and new techniques, aconitine can also be adequately processed to reduce its toxic effects and play better pharmacological effects. In our present review, we describe the toxicological effects of aconitine and further analyse aconitine poisoning cases in forensic practice. Further assessment of the underlying pharmacological properties and its safety profile are also required for better evaluation of its potential for clinical applications in future.
